# Validating 10-joint juvenile arthritis disease activity score cut-offs for disease activity levels in non-systemic juvenile idiopathic arthritis

**DOI:** 10.1136/rmdopen-2018-000888

**Published:** 2019-04-24

**Authors:** Maria Backström, Pirjo Tynjälä, Kristiina Aalto, Minna-Maija Grönlund, Heikki Ylijoki, Anne Putto-Laurila, Johanna Kärki, Paula Keskitalo, Sirja Sard, Heini Pohjankoski, Maiju Hietanen, Silke Witter, Helena Lehto, Eliisa Löyttyniemi, Paula Vähäsalo

**Affiliations:** 1 Pediatric Department, Vaasan keskussairaala, Vaasa, Finland; 2 Department of Pediatrics, South Karelia Central Hospital, Lappeenranta, Finland; 3 Poison Information Center, University of Helsinki Hospital, Helsinki, Finland; 4 Hospital for Children and Adolescents, University of Helsinki Hospital for Children and Adolescents, Helsinki, Finland; 5 Department of Pediatrics, Turku University Hospital, Turku, Finland; 6 Department of Pediatrics, Satakunta Central Hospital, Pori, Finland; 7 Department of Pediatrics, Kanta-Häme Central Hospital, Hämeenlinna, Finland; 8 Department of Paediatrics, Oulu University Hospital, Oulu, Finland; 9 Medical Research Center Oulu, Oulu University Hospital and PEDEGO Research Unit, University of Oulu, Oulu, Finland; 10 Department of Pediatrics, Päijät-Häme Central Hospital, Lahti, Finland; 11 Department of Pediatrics, Central Finland Hospital District, Jyvaskyla, Finland; 12 Department of Biostatistics, University of Turku, Turku, Finland

**Keywords:** juvenile idiopathic arthritis, disease activity, outcomes research

## Abstract

**Objectives:**

To validate cut-offs of the Juvenile Arthritis Disease Activity Score 10 (JADAS10) and clinical JADAS10 (cJADAS10) and to compare them with other patient cohorts.

**Methods:**

In a national multicentre study, cross-sectional data on recent visits of 337 non-systemic patients with juvenile idiopathic arthritis (JIA) were collected from nine paediatric outpatient units. The cut-offs were tested with receiver operating characteristic curve-based methods, and too high, too low and correct classification rates (CCRs) were calculated.

**Results:**

Our earlier presented JADAS10 cut-offs seemed feasible based on the CCRs, but the cut-off values between low disease activity (LDA) and moderate disease activity (MDA) were adjusted. When JADAS10 cut-offs for clinically inactive disease (CID) were increased to 1.5 for patients with oligoarticular disease and 2.7 for patients with polyarticular disease, as recently suggested in a large multinational register study, altogether 11 patients classified as CID by the cut-off had one active joint. We suggest JADAS10 cut-off values for oligoarticular/polyarticular disease to be in CID: 0.0–0.5/0.0–0.7, LDA: 0.6–3.8/0.8–5.1 and MDA: >3.8/5.1. Suitable cJADAS10 cut-offs are the same as JADAS10 cut-offs in oligoarticular disease. In polyarticular disease, cJADAS10 cut-offs are 0–0.7 for CID, 0.8–5.0 for LDA and >5.0 for MDA.

**Conclusion:**

International consensus on JADAS cut-off values is needed, and such a cut-off for CID should preferably exclude patients with active joints in the CID group.

**Key messages**

**What is already known about this subject?**

- Cut-off values of Juvenile Arthritis Disease Activity Score (JADAS) and clinical JADAS are useful tools for determining disease activity levels.

**What does this study add?**

- The optimal JADAS10 cut-off value for remission is 0.5 in patients with oligoarticular disease and 0.7 in patients with polyarticular disease.
- These JADAS10 cut-off values for remission excludes all patients with active joints.

**How might this impact on clinical practice?**

- JADAS10 cut-off values allow patients to understand the meaning of disease activity by providing comprehensible information in the terms: clinically inactive disease, low, moderate and high disease activity.
- The feasability of JADAS10 cut-off values would improve if a consensus could be reached.

## Introduction

Juvenile idiopathic arthritis (JIA) is a group of childhood arthritides in which prolonged synovial inflammation can lead to long-term consequences, such as growth impairment, joint destruction, osteoporosis and chronic pain.^1^ There is growing evidence that early aggressive treatment improves the outcome.^2–6^ The ultimate treatment goal is clinically inactive disease (CID),^7 8^ but this is not always achievable. There have been attempts to divide disease activity into different levels based on clinical criteria in order to develop tools to establish guidelines for therapeutic interventions, to monitor disease status in a single patient or different patient groups, and to enable comparison in research.^9–13^ The Wallace preliminary criteria of CID^7^ have further been evolved to the American College of Rheumatology (ACR) provisional criteria of CID,^8^ which also include duration of morning stiffness. The Wallace preliminary definition of CID^7^ and the ACR provisional criteria of CID^8^ have been used consistently in paediatric research, but with some controversy, the literature has shown several clinical definitions for minimal or low disease activity (LDA), moderate disease activity (MDA) and high disease activity (HDA).^9–13^ In particular, the definition of HDA differs markedly depending on which source is used.^10–14^

Some of the existing clinical criteria for disease activity levels^9 10^ are time-consuming to interpret, whereas the Juvenile Arthritis Disease Activity Score (JADAS)^15^ and especially the clinical JADAS (cJADAS) indexes^16 17^ are more useful in daily practice. The JADAS is a continuous disease activity score specific to JIA and consists of four parameters: active joint count (AJC), physician’s global assessment of disease activity, parent/patient evaluation of the child’s overall well-being and erythrocyte sedimentation rate (ESR).^15^ The cJADAS is JADAS excluding ESR.^16^ These JADAS indexes create consistency in disease activity evaluation between physicians and allow patients to understand the meaning of disease activity by providing a single score number. Nevertheless, to assess the meaning of a single JADAS score might be cumbersome. Thus, cut-off values of JADAS,^12 13 18–20^ and cJADAS,^13 17 19 20^ have been established for disease activity levels. Some variation exists in the proposed cut-off values since different references have been used.^9 11 12 15 17^ Especially the latest proposed cut-offs by Consolaro *et al* are higher than the earlier ones and in their work expert subject opinions are used as reference.^19^ Consensus on which of these cut-off values to use does not yet exist.

Recently, we established the JADAS10 and cJADAS10 intervals for the Wallace preliminary criteria of CID^7^ and LDA and MDA according to Beukelman *et al*^10 20^ and HDA according to Backström *et al*.^13^ These JADAS10 cut-off values needed validation.

### Aim

The aim was to validate the previously established JADAS10 and cJADAS10 cut-off values^13 20^ and compare them with the cut-off values suggested by Consolaro *et al*.^12 17–19^

## Methods

### Patients and statistics with statistical analyses

In Finland, nine paediatric clinics treating patients with JIA participated in the study. Patients with systemic JIA were excluded. To validate the JADAS10 and cJADAS10 cut-off levels for LDA and MDA, we used a cross-sectional method to retrospectively collect data from the most recent visit of all patients with non-systemic JIA seen during the period February 2014 to February 2016. The most recent visit of all patients resulted in a mixture of patients with varying disease activity and duration. In this way, the population consisted both of newly diagnosed and newly relapsed patients as well as patients who had been treated for several years. The population in our work defining CID, LDA and MDA cut-offs^20^ is completely different from the one in the present work. Regarding the HDA cut-offs 13 out of 14 patients with HDA in the present work are also contributing in the work where we define clinical criteria for HDA.^13^ In the work defining clinical criteria for HDA, we used the patient’s first visit^13^ and in this work we used the patient’s most recent visit.

Data were obtained on age, gender, category of JIA, AJC, ESR, physician’s global assessment of disease activity using a 10 cm linear visual analogue scale (VAS), parent/patient global assessment of well-being using a 10 cm linear VAS, and the results of rheumatoid factor levels, duration of disease and the state of uveitis (the number of cells measured in the worse eye during the most recent slit lamp examination). Additionally, data on the initiation of synthetic or biological disease-modifying anti-rheumatic drugs and both systemic and intra-articular corticosteroids at this visit were recorded. At the time point for collection of the data, the co-authors from the contributing medical centres had access to all patient data from time of diagnosis onward. The co-authors were asked to assign the patients to either an oligoarticular or polyarticular disease course based on their whole medical history of affected joints (≤4 or>4, respectively). We reported JADAS10 scores due to the fact that JADAS27 excludes clinically significant joints (eg, midtarsal and temporomandibular joints) and with JADAS10 the AJC will have the same weight as physician’s global assessment of disease activity, parent/patient global assessment of well-being and ESR.

We divided patients into four groups based on disease activity levels: CID according to the Wallace preliminary criteria,^7^ LDA and MDA according to Beukelman *et al*,^10^ and HDA according to Backström *et al*.^13^ At the time we started our first study on JADAS cut-off values in January 2013, no other clinical definition for MDA or HDA existed but the Beukelman’s definition.^10^ That is why we chose to use the Beukelman’s definition of disease activity levels and not the one by Magni-Manzoni *et al*.^9^ CID was defined according to the Wallace preliminary criteria^7^ because the duration of morning stiffness was unknown. We then validated the previously determined JADAS10 and cJADAS10 cut-off values^13 19^ and compared our cut-offs to those suggested by Consolaro *et al*.^12 17–19^

### Statistics

Originally, data from 384 patients were collected. Complete data of the core set variables were available from 337 of 384 patients. Those patients with incomplete data of the core set variables were excluded from the analysis of validation. The differences in the core set criteria of JADAS between the patients with a complete data set and those with some core set criteria missing were compared using the Mann-Whitney U test. This test was used due to the skewed distributions.

In the patients with complete data set, we determined JADAS10 and cJADAS10 cut-off values for the Wallace preliminary criteria of CID,^7^ LDA and MDA according to Beukelman *et al*,^10^ and HDA according to Backström *et al*^13^ using three different receiver operating characteristics curve (ROC)-based methods: the one closest to point (0.1) and the Youden index,^21^ which yield the highest degree of combined sensitivity and specificity, and the 75th centile. The area under the ROC curve was determined. The cut-off values were further validated by counting the correct classification rate (CCR) and the too high and too low classification rates of the values.

Analyses were performed using SPSS Statistics, V.20 (IBM, Armonk, NY, USA) and SAS System for Windows, V.9.4 (SAS Institute, Cary, NC, USA).

### Ethics

The study complies with the Declaration of Helsinki. The retrospective data were gathered without patient identification. Based on the Finnish ethical regulations, no patient consent or ethical committee approval was needed. Instead, permission was obtained from the head physicians of the hospitals participating in this study.

## Results

For the validation of earlier JADAS10 cut-off values,^20^ 384 registered most recent outpatient visits were reported. The earlier cut-off values are in oligoarticular/polyarticular disease as following; CID: 0–0.5/0.0–0.7, LDA: 0.6–2.7/0.8–3.9, MDA: 2.8–6.6/4.0–15.2 and HDA: ≥6.7/15.3. The cJADAS10 cut-off values are the same as JADAS10 cut-off-values except for HDA in polyarticular disease that is ≥14.1.^13 20^ Of the 337 patients with complete data, the median age was 9.8 (range 1.9–18.0) years, and 209 (62.0%) were girls (table 1). According to the Wallace preliminary criteria,^7^ 158 (46.9%) were classified as CID, and according to Beukelman *et al*,^10^ 76 (22.6%) were classified as LDA, 102 (30.3%) as MDA and 1 (0.3%) as HDA. Using the HDA definition proposed by Backström *et al*,^13^ 89 (26.4%) patients were classified as MDA and 14 (4.2%) as HDA. At the most recent visit, oligoarticular disease course was detected in 183 patients (54.3%) and polyarticular course in 154 (45.7%). In those with some core set criteria of JADAS missing (n=47), the data on AJC were missing in two patients, ESR in 22 patients, physician’s VAS in 26 patients and parent/patient VAS in 36 patients. Those with some core set criteria of JADAS missing had slightly more active joints (median 1 vs 0, p=0.02), had shorter disease duration (median 0.84 vs 1.44 years, p<0.0001) and were younger (median 5.9 vs 9.8 years, p=0.002) at the last recorded visit than those with a complete data set. Between groups with all versus some missing data, no difference was observed in ESR, physician’s VAS or parent/patient VAS.

**Table 1:** Clinical characteristics in a Finnish cohort of 337 patients with non-systemic JIA

Females, n (%)	209 (62.0)
Age in years, median (range)	9.8 (1.9–18.0)
Disease duration in years, median (range)	2 (0–7)
Categories of JIA	
Oligoarthritis, persistent, n (%)	155 (46.0)
Oligoarthritis, extended, n (%)	9 (2.7)
Polyarthritis, RF negative, n (%)	124 (36.8)
Polyarthritis, RF positive, n (%)	16 (4.7)
Enthesitis-related arthritis, n (%)	12 (3.6)
Psoriatic arthritis, n (%)	9 (2.7)
Undifferentiated arthritis, n (%)	4 (1.2)
Physician’s global assessment of disease activity in cm, median (IQR)	0.0 (0–1.2)
Parent/patient global assessment of well-being in cm, median (IQR)	0.5 (0–2.2)
Number of joints with active arthritis, median (IQR)	0 (0–1)
ESR in mm/h, median (IQR)	5 (2–8)
JADAS, median (IQR)	1.7 (0–4.5)
cJADAS, median (IQR)	1.6 (0–4.4)

ESR, erythrocyte sedimentation rate; JADAS, Juvenile Arthritis Disease Activity Score; JIA, juvenile idiopathic arthritis; RF, rheumatoid factor; cJADAS, clinical JADAS.

The JADAS10 and cJADAS10 cut-off values obtained through all the used ROC-based methods (table 2) were rather close to cut-off values we wanted to validate.^13 20^ Raising the JADAS10 cut-off value between CID and LDA to 1.0 in both oligoarthritis and polyarthritis, as suggested by Consolaro *et al*,^18^ lowered the CCR of LDA and led to an increase in the number of patients with LDA classified as having CID (table 3). Three of the 12 patients with oligoarticular disease whose JADAS10 was 0.6–1.0 were misclassified as having CID although they had LDA. This was due to one active joint in one patient and a physician’s VAS score of 1 in two patients. Four of the 12 patients with polyarticular disease with JADAS10 of 0.8–1.0 were misclassified as having CID although they had LDA. This was due to a physician’s VAS score of 1 in three patients and an ESR elevation of 22 in one patient.

**Table 2:** The CCR, too low and too high classification rate of JADAS10 and cJADAS10 cut-off values for selected disease activity levels of JIA, CID according to Wallace,^7^ LDA and MDA as proposed by Beukelman *et al*^10^ and HDA as proposed by Backström *et al*^13^ using three different receiver operating characteristic curve-based methods: the one closest to point (0.1), the Youden index^21^ and the 75th centile

	Cut-off range		CCR (%)		Too low (%)		Too high (%)	
	JADAS10	cJADAS10	JADAS10	cJADAS10	JADAS10	cJADAS10	JADAS10	cJADAS10
**Oligoarticular disease**								
CID^7^ (n=86)								
Closest to point	≤0.5	≤0.5	74.4	74.4	–	–	25.6	25.6
Youden index	≤0.3	≤0.3	68.6	68.6	–	–	31.4	31.4
75th centile	≤0.6	≤0.6	76.7	76.7	–	–	23.3	23.3
LDA^10^ (n=43)								
Closest to point	0.6–3.6	0.6–3.6	62.8	62.8	27.9	27.9	9.3	9.3
Youden index	0.4–3.8	0.4–3.8	83.7	83.7	11.6	11.6	4.7	4.7
75th centile	0.7–2.8	0.7–2.8	48.8	48.8	27.9	27.9	23.3	23.3
MDA^10^ (n=46)								
Closest to point	3.7–7.8	3.7–7.8	67.4	65.2	19.6	21.7	13.0	13.1
Youden index	3.9–7.8	3.9–7.8	59.0	59.0	28.0	28.0	13.0	13.0
75th centile	2.9–6.7	2.9–6.7	65.2	63.0	10.9	13.0	23.9	24.0
HDA^13^ (n=8)								
Closest to point	≥7.9	≥7.9	62.5	62.5	37.5	37.5	–	–
Youden index	≥7.9	≥7.9	62.5	62.5	37.5	37.5	–	–
75^th^ centile	≥6.8	≥6.8	75.0	75.0	25.0	25.0	–	–
Average CCR of all disease activity levels (n=154)								
Closest to point			66.8	66.3				
Youden index			68.4	68.4				
75th centile			66.4	65.9				
**Polyarticular disease**								
CID^7^ (n=72)								
Closest to point	≤1.2	≤1.2	76.4	76.4	–	–	23.6	23.6
Youden index	≤0.7	≤0.7	65.3	65.3	–	–	34.7	34.7
75th centile	≤1.0	≤1.0	76.4	76.4	–	–	23.6	23.6
LDA^10^ (n=33)								
Closest to point	1.3–5.0	1.3–4.8	69.7	66.7	24.2	24.2	6.1	9.1
Youden index	0.8–5.1	0.8–5.0	81.8	81.8	12.1	12.1	6.1	6.1
75th centile	1.0–3.8	1.0–3.8	54.5	54.5	21.2	21.2	24.3	24.3
MDA^10^ (n=43)								
Closest to point	5.1–16.0	4.9–16.0	72.0	74.4	23.3	23.3	4.7	2.3
Youden index	5.2–16.0	5.1–16.0	69.7	69.8	25.6	27.9	4.7	2.3
75th centile	3.9–11.0	3.9–11.0	55.8	55.8	20.9	20.9	23.3	23.3
HDA^13^ n=6								
Closest to point	≥16.0	≥16.0	83.3	83.3	16.7	16.7	–	–
Youden index	≥16.0	≥16.0	83.3	83.3	16.7	16.7	–	–
75th centile	≥11.0	≥11.0	100.0	100.0	0.0	0.0	–	–
Average CCR of all disease activity levels (n=154)								
Closest to point			75.4	75.2				
Youden index			75.0	75.1				
75th centile			71.7	71.7				

CCR, correct classification rate; CID, clinically inactive disease; HDA, high disease activity; JADAS, Juvenile Arthritis Disease Activity Score; JIA, juvenile idiopathic arthritis; LDA, low disease activity; MDA, moderate disease activity; cJADAS, clinical JADAS.

**Table 3:** CCR, too low and too high classification rate (%) and average CCR of JADAS10 and cJADAS10 cut-off values proposed by Backström *et al*^13 20^ and by Consolaro *et al*^12 17–19^ for selected disease activity levels of JIA; CID according to Wallace,^7^ LDA and MDA as proposed by Beukelman *et al*^10^ and HDA as proposed by Backström *et al*^13^

	Cut-off range		CCR (%)		Too low (%)		Too high (%)	
	JADAS10	cJADAS10	JADAS10	cJADAS10	JADAS10	cJADAS10	JADAS10	cJADAS10
**Oligoarticular disease**								
CID^7^								
Backström *et al*^20^	0.5	0.5	74.4	74.4	–	–	25.6	25.6
Consolaro *et al*^17 18^	1.0	1.0	84.9	84.8	–	–	15.1	15.1
Consolaro *et al*^19^	1.5	1.2	90.7	84.9	–	–	9.3	15.1
LDA^10^								
Backström *et al*^20^	0.6–2.7	0.6–2.7	41.9	41.9	27.9	27.9	30.2	30.2
Consolaro *et al*^17 18^	1.1–2.0	1.1–1.5	23.2	11.6	34.9	34.9	41.9	53.5
Consolaro *et al*^19^	1.6–3.9	1.3–3.4	48.8	48.8	46.5	37.2	4.6	14.0
MDA^10^								
Backström *et al*^13 20^	2.8–6.6	2.8–6.6	65.2	60.9	9.7	13.0	26.1	26.1
Consolaro *et al*^12 17 18^	2.1–4.2	1.6–4.0	32.6	32.6	2.2	2.2	65.2	65.2
Consolaro *et al*^19^	4.0–16.4	3.5–14.3	71.7	78.3	28.3	19.6	0.0	2.2
HDA^13^								
Backström *et al*^13^	>6.6	>6.6	75.0	75.0	25.0	24.0	–	–
Consolaro *et al*^12 18^	>4.2	>4.0	100.0	87.5	0	12.5	–	–
Consolaro *et al*^19^	>16.4	>14.3	0.0	0.0	100.0	100.0	–	–
Average CCR of all disease activity levels								
Backström *et al*^13 20^			64.1	63.1				
Consolaro *et al*^12 17 18^			60.2	54.1				
Consolaro *et al*^19^			52.8	53.0				
**Polyarticular disease**								
CID^7^								
Backström *et al*^20^	0.7	0.7	65.3	65.3	–	–	34.7	34.7
Consolaro *et al*^17 18^	1.0	1.0	76.4	76.4	–	–	23.6	23.6
Consolaro *et al*^19^	2.6	2.4	90.3	86.1	–	–	9.7	13.9
LDA^10^								
Backström *et al*^20^	0.8–3.9	0.8–3.9	63.6	63.6	12.1	12.1	24.2	24.2
Consolaro *et al*^17 18^	1.1–3.8	1.1–2.5	54.5	30.3	21.5	21.2	24.2	48.5
Consolaro *et al*^19^	2.7–5.1	2.5–5.1	39.4	42.4	54.5	51.5	6.1	6.1
MDA^10^								
Backström *et al*^13 20^	4.0–15.2	4.0–14.0	69.8	69.8	20.9	20.9	9.3	9.3
Consolaro *et al*^12 17 18^	3.9–10.5	2.6–8.5	53.4	44.2	21.0	11.6	25.6	44.2
Consolaro *et al*^19^	5.2–18.9	5.1–19.0	74.4	72.1	25.6	27.9	0.0	0.0
HDA^13^								
Backström *et al*^13^	>15.2	>14.0	100.0	100.0	0.0	0.0	–	–
Consolaro *et al*^12 18^	>10.5	>8.5	100.0	100.0	0.0	0.0	–	–
Consolaro *et al*^19^	>18.9	>19.0	83.3	50.0	16.7	50.0	–	–
Average CCR of all disease activity levels n=154								
Backström *et al*^13 20^			74.7	74.7				
Consolaro *et al*^17 18^			71.1	62.7				
Consolaro *et al*^19^			71.9	62.7				

CCR, correct classification rate; CID, clinically inactive disease; HDA, high disease activity; JADAS, Juvenile Arthritis Disease Activity Score; JIA, juvenile idiopathic arthritis; LDA, low disease activity; MDA, moderate disease activity; cJADAS, clinical JADAS.

When the JADAS10 cut-off value for CID was raised according to the newest proposal of 1.5 for patients with oligoarticular disease and 2.6 for patients with polyarticular disease,^19^ 179 patients (53.1%) were classified as being in remission (table 3), and 11 of these 179 patients (6.1%) had one active joint.

When using the cut-off value for CID of 0.5 for patients with oligoarticular disease and 0.7 for patients with polyarticular disease none of the patients classified as being in remission had any active joints (table 4).

**Table 4:** Median of the JADAS10 parameters AJC, physician’s VAS, parent/patient VAS and ESR in patient classified as CID, LDA, MDA and HDA according to cut-off values presented in the present study and those proposed earlier by Consolaro *et al*^12 18 19^

Oligoarticular disease	AJC median (range)	Parent/patient VAS median (range)	Physician’s VAS median (range)	ESR median (range)
CID				
JADAS10 ≤0.5 (n=77)	0 (0)	0.0 (0.0–0.5)	0.0 (0.0–1.0)	5 (1–20)
JADAS10 ≤1.0^18^ (n=89)	0 (0–1)	0.0 (0.0–1.0)	0.0 (0.0–1.0)	5 (1–20)
JADAS10 ≤1.5^19^ (n=99)	0 (0–1)	0.0 (0.0–1.5)	0.0 (0.0–1.0)	5 (1–20)
LDA				
JADAS10 0.6–3.8 (n=60)	0 (0–2)	0.0 (0.0–3.6)	0.5 (0.0–3.0)	4 (2–21)
JADAS10 1.1–2.0^18^ (n=17)	0 (0–1)	1.0 (0.0–2.0)	0.2 (0.0–1.0)	4 (2–11)
JADAS10 1.6–3.9^19^ (n=38)	0 (0–2)	1.5 (0.0–3.6)	0.8 (0.0–3.0)	4 (2–21)
MDA				
JADAS10 3.9–6.6 (n=28)	1 (0–3)	2.1 (0.0–5.4)	1.6 (0.0–3.0)	8 (2–31)
JADAS10 2.1–4.2^18^ (n=35)	0 (0–2)	1.6 (0.0–4.0)	1 (0.0–3.0)	4 (2–21)
JADAS10 4.0–16.4^19^ (n=46)	1 (0–3)	3.2 (0.0–10.0)	1.9 (0.0–5.0)	5 (2–31)
HDA				
JADAS10 >6.6 (n=18)	1 (0–3)	5.2 (2.6–10.0)	2.0 (1.0–5.0)	4 (2–25)
JADAS10 >4.2^18^ (n=42)	1 (0–3)	3.6 (0.0–10.0)	1.9 (0.0–5.0)	5 (2–31)
JADAS10 >16.4^19^ (n=0)				
Polyarticular disease				
CID				
JADAS10 ≤0.7 (n=52)	0 (0)	0.0 (0.0–0.6)	0.0 (0.0–1.0)	5 (1–18)
JADAS10 ≤1.0^18^ (n=64)	0 (0)	0.0 (0.0–1.0)	0.0 (0.0–1.0)	5 (1–22)
JADAS10 ≤2.6^19^ (n=89)	0 (0–1)	0.0 (0.0–2.6)	0.0 (0.0–2.0)	5 (1–33)
LDA				
JADAS10 0.8–3.9 (n=60)	0 (0–3)	1.0 (0.0–4.0)	0.5 (0.0–2.0)	5 (1–33)
JADAS10 1.1–3.8^18^ (n=39)	0 (0–2)	1.4 (0.0–3.7)	0.5 (0.0–2.0)	5 (1–33)
JADAS10 2.7–5.1^19^ (n=23)	1 (0–3)	1.7 (0.0–4.4)	1.0 (0.0–2.0)	5 (2–21)
MDA^10^				
JADAS10 4.0–15.2 (n=32)	3 (0–8)	3.8 (0.0–7.8)	2.5 (0.0–4.0)	8 (1–33)
JADAS10 3.9–10.5^18^ (n=34)	2 (0–6)	2.6 (0.0–7.8)	2.0 (0.0–4.0)	7 (1–33)
JADAS10 5.1–18.9^19^ (n=37)	3 (0–10)	4.0 (0.0–8.5)	2.5 (0.0–5.0)	1 (1–44)
**HDA****13**				
JADAS10 >15.2 (n=10)	10 (3–15)	4.6 (2.5–8.5)	4.2 (3.0–8.0)	8 (2–62)
JADAS10 >10.5^12 18^ (n=17)	7 (2–15)	4.3 (1.5–8.5)	3.0 (2.0–8.0)	7 (2–62)
JADAS10 >18.9^19^ (n=5)	10 (7–15)	4.3 (2.6–7.6)	6.5 (3.0–8.0)	16 (3–62)

AJC, active joint count; CID, clinically inactive disease; ESR, erythrocyte sedimentation rate; HDA, high disease activity; JADAS, Juvenile Arthritis Disease Activity Score; LDA, low disease activity; MDA, moderate disease activity; VAS, visual analogue scale.

In patients with oligoarticular disease, the CCR for LDA based on our earlier cut-off values^20^ was 41.9% (table 3). According to clinical criteria,^10^ as many as 35.0% of patients with LDA were classified as having CID. They were misclassified due to active uveitis and an only slightly elevated physician’s VAS score (0.1–0.5). On the other hand, 25.6% of all patients with oligoarticular disease with CID, according to the Wallace preliminary criteria,^7^ had JADAS10 scores corresponding to LDA or MDA due to high parent/patient VAS in patients with no evident signs of clinical arthritis. The JADAS10 range for LDA derived through the Youden^21^ method (JADAS10 0.4–3.8 in oligoarthritis and 0.7–5.1 in polyarthritis) had the best CCRs for LDA, which were 83.7% in oligoarthritis and 81.8% in polyarthritis. Raising our cut-off values between LDA and MDA to 3.8 in oligoarthritis and 5.1 in polyarthritis resulted in a CCR for LDA of 67.4% and 81.8%, respectively, without a negative impact on the average CCR of all activity levels (65.6% in oligoarthritis and 78.1% in polyarthritis).

The JADAS10 and cJADAS10 cut-off values for HDA in both oligoarticular and polyarticular disease were only slightly different from our earlier cut-off values (table 2),^13^ but since only 14 patients were in HDA, this result could not be used in the validation

The new proposed and validated JADAS10 cut-off values in oligoarticular/polyarticular disease are in CID: 0.0–0.5/0.0–0.7, LDA: 0.6–3.8/0.8–5.1 and MDA >3.8/5.1. Suitable cJADAS10 cut-offs are the same as JADAS10 cut-offs in oligoarticular disease. In polyarticular disease, cJADAS10 cut-offs are 0–0.7 for CID, 0.8–5.0 for LDA and >5.0 for MDA.

## Discussion

The JADAS10 cut-off values for CID, LDA and MDA established earlier^20^ have now been validated. Based on the average CCRs for all disease activity levels, cut-offs validated in this study seemed reasonable. The suggested JADAS10 cut-offs between CID and LDA in our earlier study^20^ and the present study differ markedly from those recently presented by Consolaro *et al*^19^ in a large multinational study. They proposed JADAS cut-off values between CID and LDA that are greater than 1.0, that is, 1.5 in oligoarthritis and 2.6 in polyarthritis, although these values enable patients with one active joint to be classified as in remission. This undermines the use of cut-off values as guidance in treatment decisions. When the newly proposed JADAS10 cut-off values for remission were applied in our population, altogether 11 patients with one active joint were classified as in remission.^19^ Furthermore, the cut-off value according to Consolaro *et al*^19^ for CID is markedly higher in polyarthritis than in oligoarthritis. We do not think that patients with polyarthritis should settle for markedly worse disease activity as their treatment target just because they started off with a greater number of active joints.

In our data, only 42% of the patients with oligoarticular disease with JADAS10 cut-off values of 0.6–2.7 and 64% of the patients with polyarticular disease with JADAS10 cut-off values of 0.8–3.9^20^ were correctly classified as having LDA. Thus, our JADAS10 and cJADAS10 cut-off values were rather unspecific tools for distinguishing patients with oligoarthritis with LDA. One reason for this was parent/patient VAS being high in patients with no evident signs of clinical arthritis. The Wallace preliminary criteria for CID^7^ do not include parent/patient VAS. A recent study pointed out the importance of the definition of CID because only 44% of 633 children with CID as defined by the Wallace preliminary criteria or JADAS cut-off value had CID according to both criteria.^22^ It has also been shown that patients classified as having CID according to the Wallace preliminary criteria but not by the cJADAS10 cut-off had poorer parent/patient reported outcome than those classified as having CID according to the cJADAS10 cut-off value.^23^

In the present study, the scores of parent/patient VAS were somewhat higher than those of physician’s VAS. The discrepancy between parent/patient and physician’s VAS has been shown previously.^24^ When a patient or parent has a component of chronic pain in addition to the burden of JIA, it may result in a high JADAS score due to a high parent/patient VAS even without objective signs of articular inflammation. Pain is one of the parameters that strongly influence a parent’s opinion of disease activity.^23^ In patients with JIA, pain scores associate strongly with depression.^25^ However, it has been shown that when restricting the parent/patient VAS from cJADAS, identification of patients in need of anti-tumour necrosis factor therapy decreases.^26^ At present, it is not possible to distinguish non-inflammatory pain from inflammatory pain. Pain scores need to be taken into account in the disease state due to the significant impact on patients’ well-being. However, our clinical experience is that parents who tend to catastrophise^27^ overestimate the disease burden and report a high score on the VAS scale regardless of the child’s well-being. We think that when analysing patient-reported outcomes, the catastrophising tendency of the parent and patient should be taken systematically into account by pain catastrophising questionnaire.^28^ This is very important since it would raise the chance of optimal intervention for example, coaching and supporting when this is needed instead of increasing anti-rheumatic medication in the absence of signs of inflammation.

Raising the cut-off value for LDA to 1.0 in both oligoarthritis and polyarthritis, as previously suggested,^18^ further lowered the CCR of LDA. Using that suggestion increased the proportion of patients with LDA who were reclassified as having CID according to JADAS10 cut-off values.

The earlier proposed LDA cut-off for cJADAS^17^ was unsuitable to our population, the CCR being only 11.4%. One explanation may be normal ESR in 94% of cases in the present study. In the present study, the Youden method^21^ had the best performance based on the CCR for LDA. Based on this method, the upper JADAS10/cJADAS10 limit for LDA would be 3.8/3.8 for oligoarthritis and 5.1/5.0 for polyarthritis. This is in line with the results of the large multinational study by Consolaro *et al*.^19^ It is probable that these are the optimal upper limits for LDA considering the average CCR of all disease activity levels in this study. The JADAS10 and cJADAS10 cut-off values for HDA in this study did not differ much from the cut-off values for HDA in our earlier study.^13^ However, 13 out of 14 patients with HDA in the present work were also contributing in the work where we defined clinical criteria for HDA.^13^ In the work defining clinical criteria for HDA, we used the patient’s first visit^13^ and in this work we used the patient’s most recent visit. This clearly undermines the reliability and value of the validation of the HDA cut-offs. Moreover, in this population, only 14 patients were in the HDA group, so we do not consider the cut-off values between MDA and HDA validated in this study. A separate study in is needed to validate the cut-off values for HDA.

The discrepancy between the studies seeking to find optimal JADAS cut-off values might be due to differences in cohorts and statistical methods chosen for the analyses. However, differences may be partly due to various definitions of disease activity levels, which are used as a reference. In the multinational study by Consolaro *et al*,^19^ the disease activity levels were not set by objective clinical criteria; instead, they were established according to the subjective opinion of the expert. Likewise, the disease activity levels set by Beukelman *et al*^10^ are not optimal since the HDA definition is set very high, they are not validated and they state that with a patient VAS of 2 already has MDA even if the physician sees no signs of disease activity. However, their strength is that they are objective criteria that can be interpreted in approximately the same way independent of the physician using them.

In the present study, the median disease duration was 2 years. Thus, the study population consisted mainly of patients in good disease control. A sample of newly diagnosed patients would not have been optimal for this validation study because the number of patients with CID would have been small. In order to ascertain an optimal variation of disease activity, the best time point for a study such as this would be 3–12 months from diagnosis. We cannot be sure that these results are relevant outside this cohort. Our proposed cut-off values have yet to be tested in a large multinational cohort with a greater variation in disease activity and where objective reference disease activity levels, preferably the same as used in this study, can be used as a reference.

In conclusion, we suggest the JADAS10 cut-off values to be for oligoarticular/polyarticular disease in CID: 0.0–0.5/0.0–0.7, LDA: 0.6–3.8/0.8–5.1 and MDA: >3.8/5.1. Suitable cJADAS10 cut-offs are the same as JADAS10 cut-offs in oligoarticular disease. In polyarticular disease, cJADAS10 cut-offs are 0–0.7 for CID, 0.8–5.0 for LDA and >5.0 for MDA. It is important to reach international consensus on JADAS cut-off values. It is preferable to choose a cut-off value for CID that excludes patients with active joints from being classified as having CID based on the JADAS value.
